# Integrin-Linked Kinase Reduces H3K9 Trimethylation to Enhance Herpes Simplex Virus 1 Replication

**DOI:** 10.3389/fcimb.2022.814307

**Published:** 2022-03-08

**Authors:** Meng-Shan Tsai, Shun-Hua Chen, Chih-Peng Chang, Yi-Ling Hsiao, Li-Chiu Wang

**Affiliations:** ^1^ Institute of Basic Medical Sciences, College of Medicine, National Cheng Kung University, Tainan, Taiwan; ^2^ Department of Microbiology and Immunology, College of Medicine, National Cheng Kung University, Tainan, Taiwan; ^3^ Center of Infectious Disease and Signaling Research, National Cheng Kung University, Tainan, Taiwan; ^4^ School of Medicine, I-Shou University, Kaohsiung, Taiwan

**Keywords:** integrin-linked kinase (ILK), herpes simplex virus 1 (HSV-1), trimethylation of histone H3 on lsyine 9 (H3K9me3), SUV39H1 histone methyltransferase, TRIM28/KAP1

## Abstract

Histone modifications control the lytic gene expression of herpes simplex virus 1 (HSV-1). The heterochromatin mark, trimethylation of histone H3 on lysine (K) 9 (H3K9me3), is detected on HSV-1 genomes at early phases of infection to repress viral gene transcription. However, the components and mechanisms involved in the process are mostly unknown. Integrin-linked kinase (ILK) is activated by PI3K to phosphorylate Akt and promote several RNA virus infections. Akt has been shown to enhance HSV-1 infection, suggesting a pro-viral role of ILK in HSV-1 infection that has not been addressed before. Here, we reveal that ILK enhances HSV-1 replication in an Akt-independent manner. ILK reduces the accumulation of H3K9me3 on viral promoters and replication compartments. Notably, ILK reduces H3K9me3 in a manner independent of ICP0. Instead, we show an increased binding of H3K9 methyltransferase SUV39H1 and corepressor TRIM28 on viral promoters in ILK knockdown cells. Knocking down SUV39H1 or TRIM28 increases HSV-1 lytic gene transcription in ILK knockdown cells. These results show that ILK antagonizes SVU39H1- and TRIM28-mediated repression on lytic gene transcription. We further demonstrate that ILK knockdown reduces TRIM28 phosphorylation on serine 473 and 824 in HSV-1-infected cells, suggesting that ILK facilitates TRIM28 phosphorylation to abrogate its inhibition on lytic gene transcription. OSU-T315, an ILK inhibitor, suppresses HSV-1 replication in cells and mice. In conclusion, we demonstrate that ILK decreases H3K9me3 on HSV-1 DNA by reducing SUV39H1 and TRIM28 binding. Moreover, our results suggest that targeting ILK could be a broad-spectrum antiviral strategy for DNA and RNA virus infections, especially for DNA viruses controlled by histone modifications.

## Introduction

Herpes simplex virus 1 (HSV-1) infects 49 to 87% of the world population (Looker et al., 2015). In Taiwan, the seropositive rate of HSV-1 is 95% in adults over 30 years old and 63% in the overall population (Shen et al., 2015). HSV-1 is transmitted by contact of abraded epithelial or mucosal tissues with contaminated tissues or body fluids. HSV-1 replicates in the periphery and disseminates *via* retrograde transport into neurons, where viral genomes are deposited to establish latency. Latent viral genomes remain dormant in cells until reactivation triggered by stress, trauma, or immunocompromised status in humans. Both primary and recurrent HSV-1 infections cause various diseases, including mild ulcers in the peripheral tissues, keratitis, and fatal encephalitis.

After HSV-1 binds and enters cells, the nucleocapsids are transported along the microtubules to uncoating at nuclear pore and initiate a cascade of lytic gene expression, including immediate-early (IE), early (E), and late genes. Viral gene transcription is regulated by modifications on histones associated with viral genomes. Repressive heterochromatin mark, trimethylation on histone H3 on lysine (K) 9 (H3K9me3) is detected on HSV-1 genomes in non-neuronal cells early after infection and (Silva et al., 2008; Johnson et al., 2014; Lee et al., 2016; Cabral et al., 2018; Merkl and Knipe, 2019; Gao et al., 2020) on in neurons during latency (Cliffe et al., 2009; Cliffe et al., 2015; Messer et al., 2015). Extensive studies have been focused on regulating H3K9 methylation since it suppresses lytic gene expression of infected cells (Liang et al., 2013; Messer et al., 2015) and viral reactivation from neurons during latency (Liang et al., 2009; Liang et al., 2013; Messer et al., 2015). HSV-1 DNA transcription and duplication occurs in the replication compartments in cell nuclei excluded from heterochromatin marks (Quinlan et al., 1984; Silva et al., 2008). To initiate lytic gene transcription, the HSV-1 tegument protein, VP16, recruits demethylases, JMJD and LSD1 (Liang et al., 2009; Liang et al., 2013), to demethylate H3K9 associated with viral promoters. The HSV-1 IE protein, ICP0 reduces H3K9 methylation by counteracting with nuclear DNA sensor IFI16 and chromatin remodeler ATRX (Johnson et al., 2014; Orzalli et al., 2016; Cabral et al., 2018; Merkl and Knipe, 2019). However, the responsible proteins and mechanisms of H3K9 trimethylation on HSV-1 infection remain elusive.

Integrin-linked kinase (ILK) binds to the cytoplasmic tail of β1-3 integrins (Hannigan et al., 1996). As an adaptor protein, ILK forms a complex with PINCH and parvin and mediates the interaction of integrins with cytoskeleton (Sakai et al., 2003; Wu, 2004; Wickstrom et al., 2010). As a serine/threonine kinase, ILK is activated by PI3K to phosphorylate Akt on serine 473 and modulate the cell survival, proliferation, differentiation, and migration (Persad et al., 2001; McDonald et al., 2008). In addition, ILK is shuttled into the nucleus to promote DNA replication or repress the cellular gene expression (Acconcia et al., 2007; Nakrieko et al., 2008). ILK has been shown to increases RNA virus infections. ILK enhances the coxsackie B3 virus and Rift Valley Fever virus infections by activating Akt (Esfandiarei et al., 2006; Pinkham et al., 2017). ILK also increases hepatitis C virus infection by suppressing type I interferon (IFN) and IFN-stimulated gene (ISG) expression (Kuwashiro et al., 2018). However, little is known about the effect of ILK on DNA virus infection. Previous studies showed that Akt is activated in HSV-1-infected cells to facilitate viral entry (Cheshenko et al., 2013; Musarrat et al., 2018) and protein translation (Chuluunbaatar et al., 2010; Norman and Sarnow, 2010), suggesting a pro-viral role of ILK in HSV-1 infection that has not been addressed before.

Here, we reveal that ILK enhanced HSV-1 replication by reducing the levels of H3K9me3 associated with HSV-1 DNA and replication compartments. In contrast to the role of ILK in RNA virus infections, ILK-mediated Akt activation was dispensable for HSV-1 replication. We demonstrate that the H3K9 methyltransferase SUV39H1 and transcriptional corepressor TRIM28 bound viral promoters and repressed lytic gene transcription. ILK antagonized the SUV39H1- and TRIM28-mediated repression by promoting TRIM28 phosphorylation on serine 473 and 824. The ILK inhibitor, OSU-T315, suppressed HSV-1 replication in cells and mice. In conclusion, we demonstrate that ILK reduces heterochromatin marks on HSV-1 DNA to elevate viral gene transcription and replication. Moreover, our findings indicate that ILK could be a potent and broad-spectrum antiviral target against DNA virus infections controlled by histone modifications.

## Materials and Methods

### Plasmids

The control (sh*LacZ*, TRCN0000072223) and ILK-specific shRNA (sh*ILK*, TRCN0000279854) targeting to *ILK* 3’-UTR in the pLKO vector were obtained from National RNAi Core Facility, Institute of Molecular Biology and Genomic Research Center, Academia Sinica, Taiwan. The pLKO vectors were packed into lentiviruses by the RNAi core laboratory in the Clinical Medicine Research Center of National Cheng Kung University Hospital, Taiwan. The pcDNA3.1(+) vector with human ILK carry 3×FLAG tag at N-terminus was constructed (Leadgene Biomedical). The E359K ILK (Persad et al., 2001; Maydan et al., 2010) was constructed *via* two-step site-directed mutagenesis and sequenced. The pGL3 with ICP4 promoter (-597 to -237 relative to the ICP4 transcription initiation site) was described previously (Chen et al., 2008). The promoter region of ICP0 (-171 to +57 relative to the ICP0 transcription initiation site) was amplified from HSV-1 DNA and cloned into pGL3 vector. Primers used in plasmid constructions are listed in **Supplementary Table 1**.

### Cells, Viruses, and Infections

The human neuroblastoma SK-N-SH cells, retinal pigment epithelial ARPE-19, osteosarcoma U2OS cells kindly provided by Dr. Chia-Yih Wang, National Cheng Kung University, and Vero cells were maintained according to the American Type Culture Collection’s instructions. HSV-1 strain KOS and KOS-derived ICP0-null virus, n212, were propagated and titrated on Vero and U2OS cell monolayers, respectively. The SK-N-SH cells and HSV-1 strain KOS were used in most experiments, otherwise specified. To establish ILK knockdown cells, SK-N-SH cells were infected with lentiviruses carrying control or ILK-specific shRNA in the medium with polybrene (Sigma-Aldrich) and selected in the medium with 0.8 mg/ml puromycin (Sigma-Aldrich). The viability of control and ILK knockdown cells and cells treated with OSU-T315 (MedChemExpress) was assayed by Cell Counting Kit-8 (Enzo Life Science). Cells were infected HSV-1 (MOI 1 to 3, unless specified) at room temperature for 45 min, washed with PBS, and incubated at 37°C until indicated time points. Infected cells were treated with indicated concentrations of OSU-T315 after 1 hour of infection. For ectopic ILK expression, cells were transfected with a control vector or vectors with wild-type or E359K ILK *via* Lipofectamine™ 3000 reagent (Thermo Fisher Scientific) for 6 hours and then infected with HSV-1 for 24 hours. For knocking down ILK, SUV39H1, or TRIM28 expression, cells were transfected with scrambled siRNA (si*SCR*) or siRNA targeting ILK, SUV39H1, or TRIM28 (Thermo Fisher Scientific) *via* Lipofectamine™ RNAiMAX (Thermo Fisher Scientific) for 24 hours and then infected with HSV-1 for indicated hours.

### Entry and Penetration Assay

The SK-N-SH monolayers were subjected for entry and penetration assay as previously described (MacLean, 1997; Fan et al., 2017; Ibanez et al., 2018). For entry assay, cells were infected with HSV-1 at room temperature for 45 min, washed with PBS to remove the unbound virus, treated with citric buffer (pH = 3) to inactivate the bound virus remained on cell surface, harvested by trypsin digestion, and subjected to quantify viral genomes entered in cells by real-time PCR. The percentage of internalized HSV-1 DNA was calculated as % of internalized DNA = (HSV-1 genomes in citric buffer-treated cells/HSV-1 genomes in PBS-treated cells) × 100. For penetration assay, monolayers of cells in 6-well plates were infected with HSV-1 (20 PFU per well) at room temperature for 45 min and washed with citric buffer (pH = 3) and PBS. The acidic citric buffer inactivates the virus bound on cell surface that does not enter. After wash, the cell monolayers were incubated in medium with methylcellulose overlay for plaque formation. HSV-1 plaques were stained and counted 3 days later.

### Real-Time PCR

Real-time PCR was performed using Fast SYBR green reagents and StepOne real-time PCR system (Thermo Fisher Scientific). Primers are listed in **Supplementary Table 1**. For determining the number of HSV-1 genomes, total DNA in cells were extracted, and the copy number of HSV-1 genomes was determined. For accessing the transcript levels of HSV-1 lytic genes and cellular genes, total RNA was extracted from mock-infected or HSV-1-infected cells at the indicated time points, reverse transcribed into cDNA, and subjected to determine the levels of viral and cellular genes. The relative HSV-1 DNA and viral and cellular RNA levels were calculated as ΔC_T_ = C_T_ target gene – C_T_ *ACTB* and ΔΔC_T_ = ΔC_T_ sample – ΔC_T_ control. Fold increase was calculated as 2^-ΔΔ^ *^CT^*.

### Western Blot Analysis

The cell pellets were lysed in lysis buffer (Cell Signaling) with a protease inhibitor cocktail (Sigma Aldrich). Total cell lysates were subjected to western blotting with antibodies against ICP0 (clone 5H7; Santa Cruz), ICP4 (clone H943; Santa Cruz), ICP8 (clone 10A3; Santa Cruz), TRIM28 with phosphorylated Ser473 (clone 11G10SC; BioLegend), TRIM28 with phosphorylated Ser824 (Abcam), TRIM28 (Sigma-Aldrich), ILK (Genetex), Akt with phosphorylated Ser473 (Genetex), Akt (Genetex), FLAG (clone M2; Merck), SUV39H1 (clone MG44; mouse ascites; Millipore), and β-actin (clone C4; Millipore) and HRP-conjugated secondary antibodies. Protein bands were developed by an enhanced chemiluminescence substrate kit (Millipore) and detected by UVP system. The intensity of the protein bands was measured by ImageJ software.

### Luciferase Assay

Uninfected cells were transfected without or with indicated siRNA for 24 hours and with pGL3 vectors carrying *ICP0* or *ICP4* promoters by Lipofectamine™ 3000 reagent for another 24 hours. The resulting cells were subjected to determine luciferase activity by a kit (Promega) according to the manufacturer’s instructions and SpetraMax i3x system (Molecular Devices).

### Chromatin Immunoprecipitation Assay (ChIP)

Total 1 × 10^7^ cells were seeded per 15-cm dish 24 hours before infection, infected with HSV-1 for 3 hours, fixed by 1% paraformaldehyde, treated with 125 mM glycine, and collected. The nuclei were released from cells by cellular lysis buffer (5 mM PIPES pH = 8, 85 mM KCl, and 0.5% NP40), pelleted, and resuspended in nuclear lysis buffer (50 mM Tris pH = 8, 10 mM EDTA, and 1% SDS). The resulted nuclear lysates were sonicated to shear DNA into lengths smaller than 1,000 b.p. An aliquot of nuclear lysates was diluted 1 ml IP dilution buffer (150 mM NaCl, 10 mM Na_2_HPO_4_, 2 mM EDTA, 1.1% Triton X100, and 0.1% SDS) and incubated with 1:200 diluted control antibodies or antibodies against H3 (Millipore), trimethyl H3K9 (clone 6F12-H4; Millipore), trimethyl H4K20 (Millipore), acetyl H3K9 (Millipore), acetyl H3K14 (Millipore), SUV39H1, SUV39H2 (Abcam), or TRIM28 at 4°C for 16 to 18 hours and then with Magna ChIP™ protein A+G magnetic beads (Millipore) at 4°C for 3 hours. Resulting beads were washed with low-salt wash buffer (150 mM NaCl, 20 mM Tris-HCl pH = 8.1, 2 mM EDTA, 1% Triton X100, 0.1% SDS, and 1 mM PMSF), LiCl wash buffer (50 mM HEPES pH 7.5, 250 mM LiCl, 1 mM EDTA, 1% NP-40, 0.7% sodium deoxycholate, and 1 mM PMSF), and Tris-EDTA buffer (10 mM Tris-HCl pH = 8 and 1 mM EDTA). The protein-DNA complex was eluted by incubating beads in elution buffer (1% SDS and 0.1 M NaHCO_3_) at 65°C for 20 min. The elution was incubated at 95°C for 30 min with a final concentration of 0.2 M NaCl to remove crosslinks. The resulting solutions were incubated with proteinase K and RNase A. The DNA fragments were purified by GenepHlow™ Gel/PCR Kit (Geneaid) and determined by real-time PCR with primers listed in **Supplementary Table 1**. Anti-H3 antibody serves as a positive control and control antibody serves as a negative control for ChIP experiments. The fold enrichment was calculated as the fraction of DNA immunoprecipitated by the specific antibody relative to the 1% input normalized to the fraction of viral DNA immunoprecipitation by control antibody relative to the 1% input in the same reaction.

### Immunofluorescence Assay

Cells were seeded on coverslips in 24-well plates for 24 hours, infected with HSV-1 for 6 hours, fixed by 4% paraformaldehyde, and permeabilized by PBS with 0.1% Triton X-100. After blocking with 5% BSA, cells were stained with antibodies against ICP8, trimethyl H3K9 (Genetex), trimethyl H4K20 (Genetex), and Alexa488- or Alexa594-conjugated secondary antibodies. Cells were mounted in medium with DAPI (Abcam). Images were obtained by Olympus FLUOVIEW FV1000 confocal microscope and analyzed by ImageJ software.

### Mouse Infection Study

The C57BL/6J mice were maintained under specific-pathogen-free conditions in the Laboratory Animal Center of National Cheng Kung University. The care and use of mice protocols were approved by the Institutional Animal Care and Use Committee in National Cheng Kung University with the approval number 108101. The ILK-inhibitor, OSU-T315, was prepared in 0.5% methylcellulose solution containing 0.1% Tween 80 with a final concentration of 0.6 or 1.2 mg/ml. The 6- to 8-week-old mice were anesthetized and infected with 2 × 10^5^ PFU of KOS or mock-infected with lysates of uninfected Vero cells topically on the eyes following scarification of the cornea with a needle 20 times. Mice were treated with 5 µl OSU-T315 topically on the infected eye and 10 µl/g OSU-T315 *via* intragastric administration daily started from 1 hour postinfection. Mouse eyes and trigeminal ganglia were harvested and subjected to determine HSV-1 titers *via* plaque assay at indicated time points.

### Statistics

Data represent the mean ± or + SEM (error bar) from at least 4 samples per data point or per group. For statistical comparisons, the levels of viral yields in cells and tissues were analyzed by Mann-Whitney *U* test, rest of the experiments were analyzed by Student *t* test. All *p* values are for two-tailed significance tests. A *p* value of < 0.05 is considered statistically significant.

## Results

### ILK Enhances HSV-1 Replication Independent of Akt Activation

The SK-N-SH human neuroblastoma cell line, which supports HSV-1 replication (Zhou et al., 2000; Zheng et al., 2014), is used to study the role of ILK in HSV-1 infection. SK-N-SH cells were stably transfected with control shRNA (sh*LacZ*) or shRNA targeting the 3’UTR of *ILK* mRNA (sh*ILK*). The ILK-specific shRNA diminished *ILK* mRNA and protein levels by 90% and 98% (**Supplementary Figures 1A, B**). Disruption of the *Ilk* gene in chondrocytes reduces cell proliferation and leads to chondrodysplasia in mice (Grashoff et al., 2003). Thus, we measured cell viability and found that ILK knockdown failed to affect cell viability and proliferation (**Supplementary Figure 1C**).

**Figure 1:**
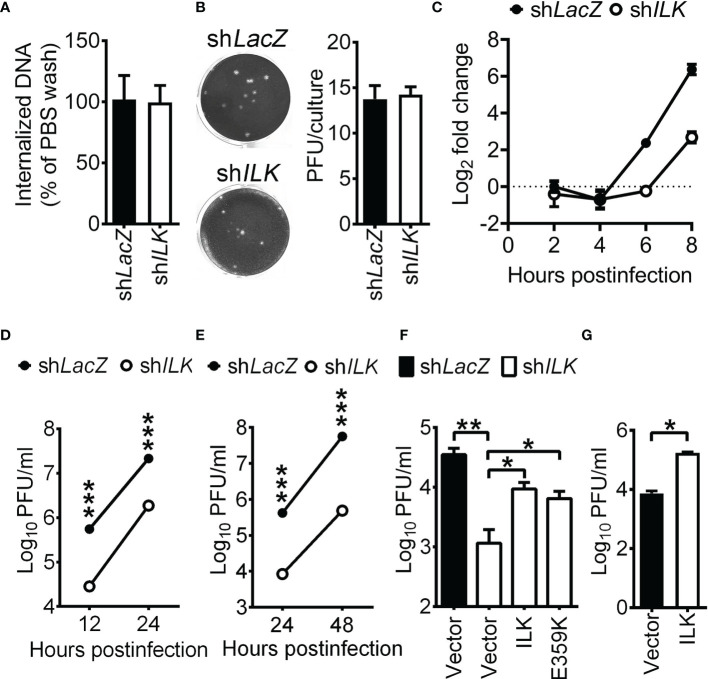
ILK enhances HSV-1 replication independent of Akt activation. **(A)** The control (sh*LacZ*) and ILK knockdown (sh*ILK*) cells were infected with HSV-1, washed with citric buffer (pH = 3) or PBS, collected after trypsinization, and subjected to determine genome numbers *via* real-time PCR. The percentage of internalized viral DNA was calculated as the number of viral genomes in citric buffer-treated cells normalized to that of PBS-treated cells. **(B)** For the penetration assay, cells were infected with HSV-1 (20 PFU) at room temperature for 45 min, washed with citric buffer (pH = 3), and incubated in the medium with methylcellulose overlay for plaque formation. The representative images (left panel) and quantitative results (right panel) are shown. **(C)** The relative HSV-1 DNA levels in control and ILK knockdown cells infected with HSV-1 at indicated hours postinfection are shown. The level of HSV-1 DNA to internal control in control cells at 2 hours postinfection was set as 1. **(D, E)** The viral yields in the control and ILK knockdown cells infected with HSV-1 at MOI of 1 **(D)** or 0.01 **(E)** at indicated hours postinfection are shown. **(F)** The viral yields in the control and ILK knockdown cells transfected with a control vector (Vector) or the vector expressing wild-type ILK (ILK) or ILK E359K mutant and infected with HSV-1 (MOI = 0.01) at 24 hours postinfection are shown. **(G)** The viral yields in the SK-N-SH cells transfected with a control vector (Vector) or the vector expressing ILK (ILK) and infected with HSV-1 (MOI = 0.01) at 24 hours postinfection are shown. Data represent mean ± or + SEM (error bar). *p < 0.05, **p < 0.01, ***p < 0.001 *via* Mann-Whitney *U* test.

ILK mediates the interaction between integrins and cytoskeleton (Wu, 2004), and ILK deficiency disrupts the actin and microtubule structure in cells (Sakai et al., 2003; Wickstrom et al., 2010). HSV-1 triggers F-actin oligomerization to facilitate viral entry (Xiang et al., 2012). Accordingly, we assessed whether HSV-1 entry into cells is reduced in ILK knockdown cells as previously described (Zheng et al., 2014; Ibanez et al., 2018). Cells were infected with HSV-1 at room temperature for 45 min. After washing with citric buffer (pH = 3), the cells were harvested by trypsin digestion to remove the virus remaining on the cell surface. The viral genomes entered cells were determined *via* quantitative real-time PCR. Room temperature, instead of 4°C, was used for study since SK-N-SH cells failed to withstand 45 min incubation at 4°C. After 45 min of infection, we found that comparable virus genomes were detected in the control and ILK knockdown cells (**Figure 1A**), showing that ILK fails to affect HSV-1 entry. We also monitored the effect of ILK on HSV-1 penetration. The control and ILK knockdown cell monolayers were infected with HSV-1 (20 PFU per culture), washed with citric buffer, and incubated in the medium with methylcellulose overlay for plaque formation. The number of plaques formed in monolayers of ILK knockdown cells was not different from that of control cells (**Figure 1B**). These results suggest that ILK enhances HSV-1 infection in the step after virus penetration. Next, we monitored if ILK knockdown decreases the levels of the viral DNA. In infected control cells, HSV-1 DNA began to duplicate after 4 hours postinfection (hpi), and ILK knockdown reduced the levels of HSV-1 DNA at 6 and 8 hpi by 4- and 8-fold, respectively, and the mean viral yields at 12 and 24 hours postinfection (**Figures 1C, D**). Similar results were observed in cells infected with HSV-1 at MOI of 0.01 (**Figure 1E**). To confirm if ILK enhances HSV-1 replication in other cell types, we attempted to establish ILK knockdown in ARPE-19 cells but failed, suggesting that ILK regulates cell survival in a manner specific to cell types. The results are consistent with previous studies showing that ILK is required for the proliferation of T cells and chondrocytes, but not adipocytes and muscles in mice (Grashoff et al., 2003; Liu et al., 2005; Kang et al., 2016; Bugler-Lamb et al., 2021). Therefore, we transfected SK-N-SH and ARPE-19 cells with specific siRNA targeting *ILK* coding sequence (si*ILK*). ILK-specific siRNA but not scrambled siRNA (si*SCR*) diminished ILK protein levels by 60% (**Supplementary Figures 2A, C**) and reduced HSV-1 yields at 24 hpi (**Supplementary Figures 2B, D**), showing that ILK is an enhancing factor for HSV-1 replication in a manner independent of cell types.

PI3K-Akt signaling has been shown to facilitate HSV-1 infection (Chuluunbaatar et al., 2010; Norman and Sarnow, 2010; Cheshenko et al., 2013; Musarrat et al., 2018), suggesting that ILK may activate Akt to enhance HSV-1 replication. To assess this possibility, we determined the levels of phosphorylated Akt on serine 473 (Ser^473^) and HSV-1 yields in ILK knockdown cells without or with reconstituted ILK expression. The ectopically expressed ILK with 3×FLAG tag is resistant to ILK-specific shRNA due to the lack of endogenous *ILK* 3’UTR. Without HSV-1 infection, the ectopic expression of ILK restored ILK expression in knockdown cells without affecting Akt Ser^473^ phosphorylation (**Supplementary Figure 3**). After 24 hours of infection, the levels of Akt Ser^473^ were increased in control cells and ILK knockdown cells without or with ectopic expression of ILK. As the remaining ILK protein in knockdown cells might result in Akt activation in response to HSV-1 infection, we reconstituted the expression of ILK with E359K mutation in knockdown cells. Previous studies showed that E359K mutant impairs ILK-mediated Akt activation in a dominant-negative manner (Persad et al., 2001; Nikolopoulos and Turner, 2002). In ILK knockdown cells, the ILK E359K mutant failed to affect Akt activation before infection and abrogated Akt activation after 24 hours of infection (**Supplementary Figure 3**). **Figure 1F** showed that ILK knockdown reduced the mean HSV-1 yields, and both wild-type and E359K ILK restored HSV-1 replication in knockdown cells, indicating that ILK promotes HSV-1 replication independent of Akt activation. The ectopic ILK expression increased HSV-1 yields in SK-N-SH cells (**Figure 1G**).

### ILK Increases HSV-1 IE and E Gene Expression by Reducing H3K9me3 on Viral Promoters

**Figure 2:**
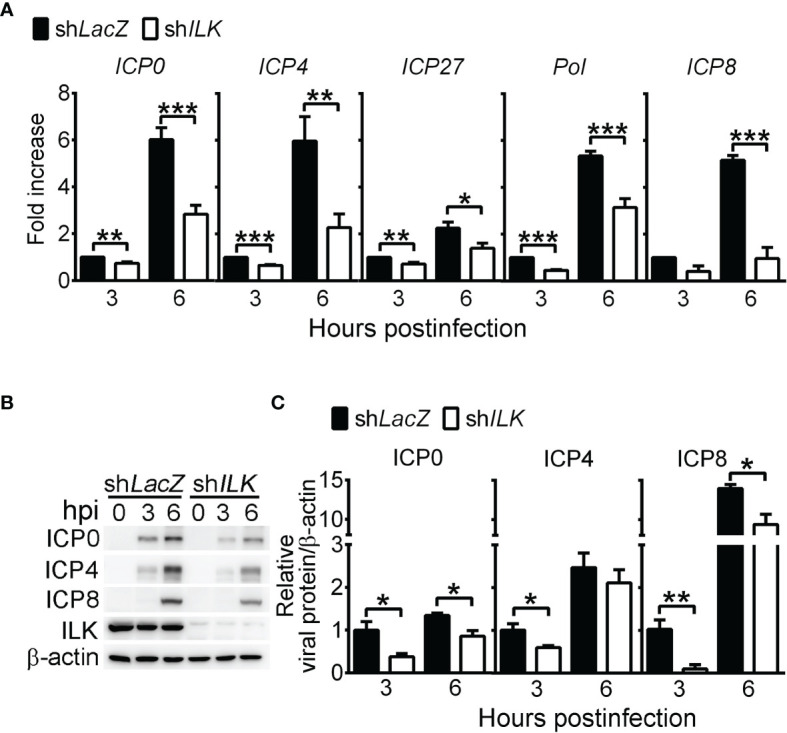
ILK increases HSV-1 IE and E gene expression. **(A)** The relative transcript levels of viral IE genes (*ICP0*, *ICP4*, and *ICP27*) and E genes (*Pol* and *ICP8*) in control (sh*LacZ*) and ILK knockdown (sh*ILK*) cells at indicated hours postinfection (hpi) quantified by real-time PCR are shown. The transcript levels of indicated genes normalized to *ACTB* mRNA in control cells at 3 hours postinfection were set as 1. **(B, C)** The representative western blots **(B)** and relative levels **(C)** of indicated proteins in control and ILK knockdown cells infected with HSV-1 at indicated hours postinfection are shown. The levels of indicated viral proteins normalized to β-actin in control cells at 3 hours postinfection were set as 1. Data represent mean + SEM (error bar). *p < 0.05, **p < 0.01, ***p < 0.001 *via* Student *t* test.

Since we found that ILK knockdown decreased HSV-1 DNA duplication, we were interested if ILK enhances viral gene expression before DNA duplication by determining the levels of viral IE genes, *ICP0*, *ICP4*, and *ICP27*, and E genes, DNA polymerase (*Pol*), and *ICP8*. ILK knockdown significantly decreased the transcript levels of viral IE and E genes at 3 and 6 hpi by 30 to 80%, except ICP8 at 3 hpi (**Figure 2A**). Similar results were observed in the corresponding protein levels. Viral proteins were detected in control cells as early as 3 hpi. ILK knockdown diminished the levels of viral proteins at 3 and 6 hpi by 20 to 95%, except ICP4 at 6 hpi (**Figures 2B, C**). These results show that ILK facilitates viral IE and E gene transcription to enhance viral replication. To investigate if ILK antagonizes type I IFN responses as previously reported in HCV infection (Kuwashiro et al., 2018), we determined the mRNA levels of *IFNB*, *CXCL10*, and *ISG15* in infected cells. Comparable levels of *IFNB*, *CXCL10*, and *ISG15* were detected in infected control and ILK knockdown cells at 6 hpi (**Supplementary Figure 4**). These results show that ILK facilitates viral gene expression without affecting type I IFN induction.

**Figure 3:**
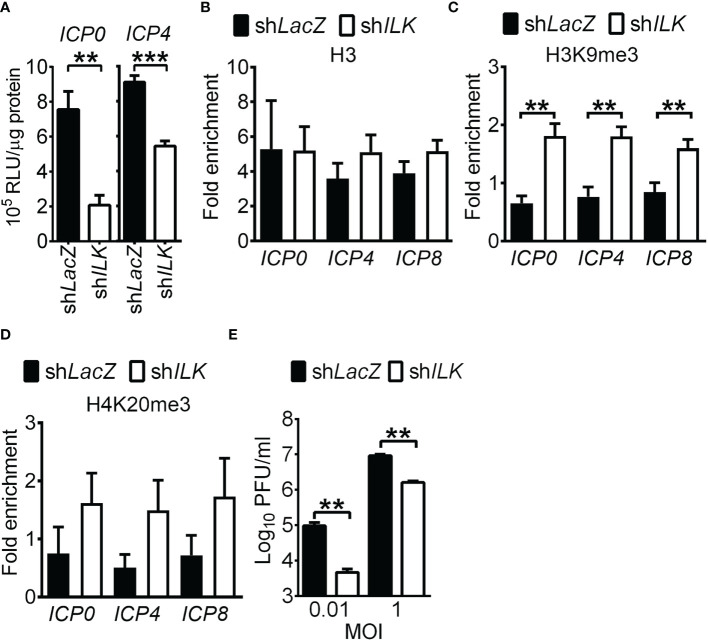
ILK reduces H3K9me3 on HSV-1 promoters in a manner independent of ICP0. **(A)** The luciferase activities in uninfected control (sh*LacZ*) and ILK knockdown (sh*ILK*) cells transfected with pGL3 vector carrying *ICP0* or *ICP4* promoters are shown. **(B–D)** The relative levels of H3 **(B)**, trimethyl (me3) H3K9 **(C)**, and H4K20me3 **(D)** associated with *ICP0*, *ICP4*, and *ICP8* promoters in control and ILK knockdown cells infected with HSV-1 at 3 hours postinfection are shown. Fold enrichment was calculated as the fraction of DNA immunoprecipitated by the specific antibody/input normalized to the control antibody/input. **(E)** The HSV-1 yields in the control and ILK knockdown cells infected with ICP0-null virus (n212) at indicated MOI after 24 hours of infection are shown. Data represent mean + SEM (error bar). **p < 0.01, ***p < 0.001 *via* Mann Whitney *U* test in panel E and *via* Student *t* test in the rest panels.

To study how ILK augments HSV-1 IE and E gene expression, we compared the activity of luciferase driven by the *ICP0* or *ICP4* promoter in uninfected control and ILK knockdown cells. ILK knockdown significantly decreased the promoter activities of *ICP0* and *ICP4* (**Figure 3A**), suggesting that ILK regulates intrinsic cellular factors to activate HSV-1 transcription. Since histone modifications control HSV-1 lytic gene transcription, we determined the levels of repressive marks, including H3K9me3 and H4K20me3, on viral *ICP0*, *ICP4*, and *ICP8* promoters using ChIP assay. We also detected the active marks, acetylation (Ac) on H3K9 and H3K14, accumulated on HSV-1 genomes in infected cells observed previously (Kulej et al., 2017). The levels of H3 associated with viral promoters were comparable in control and ILK knockdown cells (**Figure 3B**), and ILK knockdown increased the levels of H3K9me3 and H4K20me3 on all three promoters (**Figures 3C, D**). There was no difference in the levels of active marks, H3K9Ac and H3K14Ac, on all three promoters in control and ILK knockdown cells (**Supplementary Figures 5A, B**). These results show that ILK promotes transcription activation by reducing H3K9me3 on HSV-1 IE and E promoters. HSV-1 ICP0 serve as transactivator to activate viral gene transcription by facilitating H3K9me3 removal (Johnson et al., 2014; Orzalli et al., 2016; Cabral et al., 2018; Merkl and Knipe, 2019). To study if ILK enhances viral IE and E gene expression *via* interacting with ICP0, we infected cells with KOS-derived ICP0-null virus, n212 (Cai and Schaffer, 1989). The mean viral yields were lower in ILK knockdown cells than in control cells infected with low (0.01) or high (1) MOI at 24 hpi (**Figure 3E**). The results show that depleting ICP0 fails to abrogate ILK-increased HSV-1 replication. Collectively, the results suggest that ILK reduces H3K9me3 accumulation on the viral genomes in a manner independent of ICP0.

**Figure 4:**
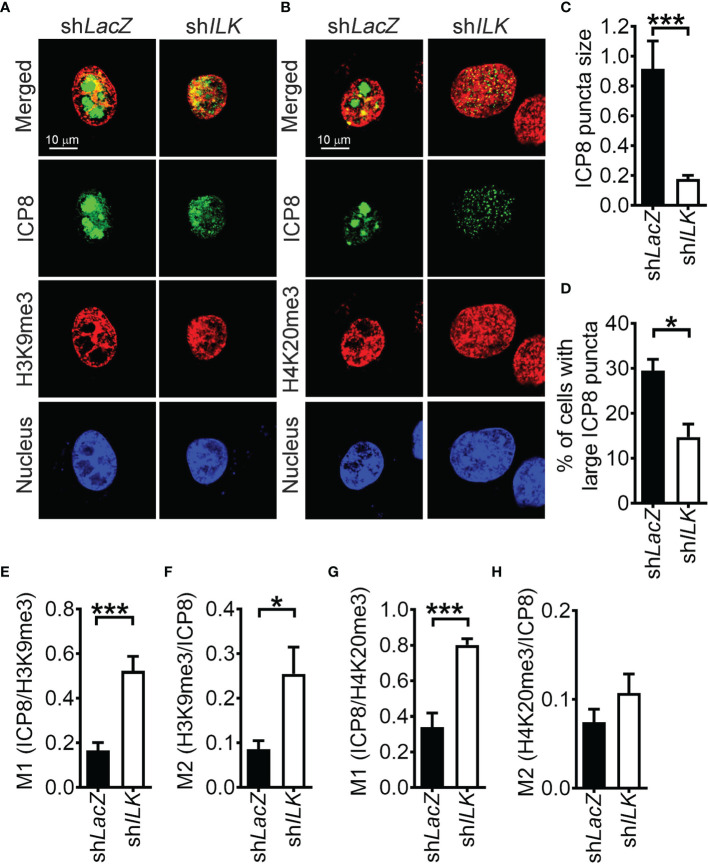
ILK promotes heterochromatin mark exclusion from HSV-1 replication compartments. **(A, B)** The representative images of control (sh*LacZ*) and ILK knockdown (sh*ILK*) cells infected with HSV-1 for 6 hours and stained with antibodies against ICP8, H3K9me3, or H4K20me3 are shown. Cell nuclei are counterstained with DAPI. **(C)** The size of ICP8 puncta was measured by particle analysis of ImageJ software. **(D)** The percentage of ICP8-positive cells with large puncta was calculated from 3 independent experiments. **(E–H)** The colocalization ICP8 with H3K9me3 or H4K20me3 was quantified by the Jacob plugin of ImageJ software as Manders’ coefficient M1 and M2. Data represent mean ± SEM (error bar). *p < 0.05, ***p < 0.001 *via* Student *t* test.

HSV-1 transcribes genes and replicates in confined regions in the nucleus called replication compartments. We found that HSV-1 gene transcription and genome duplication were impaired in ILK knockdown cells. Hence, it is of our interest to determine whether ILK knockdown affects the replication compartment formation. At 3 hpi, small ICP8-positive puncta were detected in a small fraction of control and ILK knockdown cells. At 6 hpi, ICP8 aggregated into large punta, forming replication compartments in control cells (**Figures 4A, B**). ILK knockdown significantly reduced the size of ICP8-positive puncta in infected cells and the percentage of infected cells with aggregated ICP8-puncta (**Figures 4C, D**). These results were consistent with our finding that the ILK knockdown suppressed viral DNA duplication and gene transcription at 6 hpi (**Figures 1C** and **2A**, respectively). Moreover, we analyzed the colocalization of ICP8 with H3K9me3 or H4K20me3 expressed as Manders’ coefficient (Dunn et al., 2011) to assess if the replication compartments are excluded from heterochromatin marks. The M1 of Manders’ coefficient indicates the fraction of ICP8 colocalized with H3K9me3 or H4K20me3 (**Figures 4E, G**), and M2 indicates the fraction of H3K9me3 or H4K20me3 colocalized with ICP8 (**Figures 4F, H**). ILK knockdown increased the colocalization of ICP8 with H3K9me3 in both cases and increased the fraction of ICP8 colocalized with H4K20me3. These results show that ILK promotes the exclusion of heterochromatin marks, especially H3K9me3, from replication compartments.

### ILK Antagonizes the TRIM28-SUV39H1-Repressed Viral Gene Transcription

**Figure 5:**
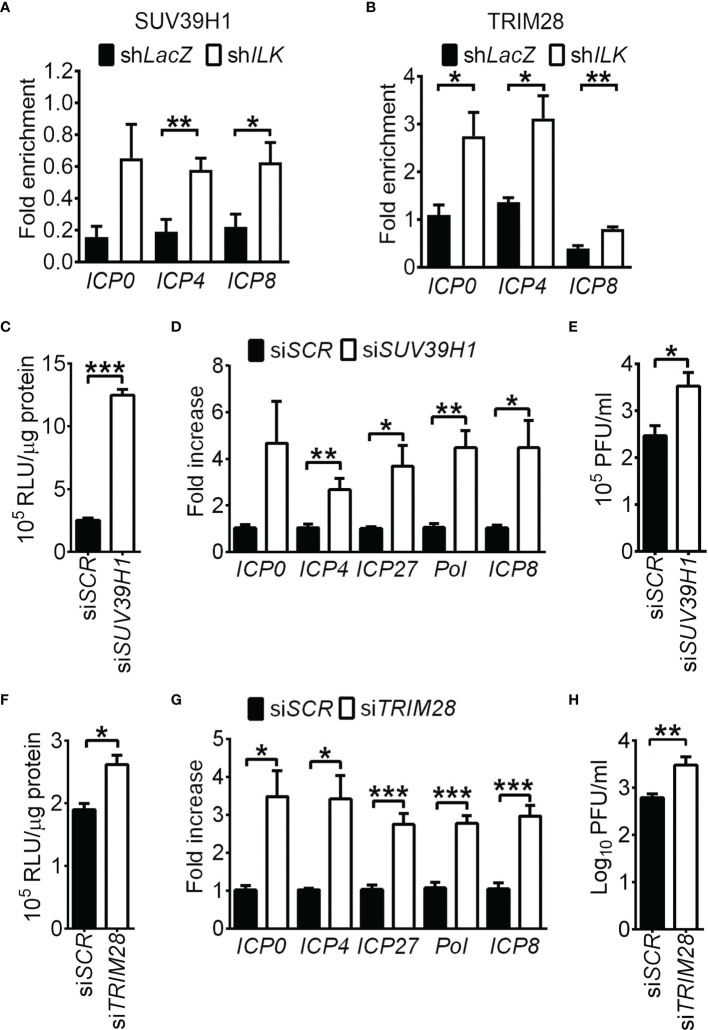
ILK antagonizes the TRIM28-SUV39H1-repressed viral gene transcription. **(A, B)** The relative levels of SUV39H1 **(A)** and TRIM28 **(B)** associated with *ICP0*, *ICP4*, and *ICP8* promoters in control (sh*LacZ*) and ILK knockdown (sh*ILK*) cells infected with HSV-1 at 3 hours postinfection are shown. Fold enrichment was calculated as the fraction of DNA immunoprecipitated by the specific antibody/input normalized to the control antibody/input. **(C, F)** The luciferase activities in uninfected ILK knockdown cells transfected with scrambled siRNA (si*SCR*) or siRNA targeting SUV39H1 (si*SUV39H1*) or TRIM28 (si*TRIM28*) and pGL3 vector carrying *ICP4* promoter are shown. **(D, G)** The relative transcript levels of indicated viral genes in ILK knockdown cells transfected with indicated siRNA and infected with HSV-1 for 6 hours are shown. The transcript levels of indicated genes relative to *ACTB* in ILK knockdown cells with si*SCR* were set as 1. **(E, H)** The viral yields in ILK knockdown cells transfected with indicated siRNA and infected with HSV-1 (MOI = 0.01) for 24 hours are shown. Data represent mean + SEM (error bar). *p < 0.05, **p < 0.01, ***p < 0.001 *via* Mann-Whitney *U* test in panel E and H and Student *t* test in the rest panels.

Although the regulation of H3K9 methylation is extensively studied in HSV-1 infection (Liang et al., 2009; Liang et al., 2013; Johnson et al., 2014; Orzalli et al., 2016; Cabral et al., 2018; Merkl and Knipe, 2019), the methyltransferase responsible for H3K9me3 in HSV-1 infection remains to be identified. SUV39H1 and SUV39H2 catalyze di- and trimethylation of H3K9 (Greer and Shi, 2012) and facilitate trimethylation on H4K20 by binding to TRIM28 and H4K20 methyltransferase SUV420H (Jang et al., 2018; Wang et al., 2019). Loss of SUV39H1 or TRIM28 abolishes trimethylation on H3K9 and H4K20 and heterochromatin formation (Schotta et al., 2004; Jang et al., 2018). Both SUV39H1 (1.0 ± 0.07) and H2 (23 ± 0.7, relative to SUV39H1) mRNA were expressed in SK-N-SH cells and remained unchanged in ILK knockdown cells (1.2 ± 0.2 and 0.9 ± 0.03 for SUV39H1 and H2 relative to control cells, respectively). We measured if ILK abolishes the binding of SUV39H1, SUV39H2, and TRIM28 to HSV-1 promoters. ILK knockdown increased the levels of SUV39H1 and TRIM28 on *ICP0*, *ICP4*, and *ICP8* promoters (**Figures 5A, B**), while the effect of ILK knockdown on SUV39H2 binding was limited (**Supplementary Figure 5C**). To confirm the repressive role of SUV39H1 and TRIM28 in HSV-1 replication, we knocked down SUV39H1 or TRIM28 expression in ILK knockdown cells *via* specific siRNA. The gene-specific siRNA (si*SUV39H1* and si*TRIM28*), but not scrambled siRNA (si*SCR*), decreased SUV39H1 and TRIM28 protein levels by 80% (**Supplementary Figures 6A, B**). Knocking down SUV39H1 in infected ILK knockdown cells increased ICP4 promoter activity (**Figure 5C**) and elevated viral gene transcription and viral yield (**Figures 5D, E**). Similar results were observed in ILK knockdown cells transfected with TRIM28-specific siRNA (**Figures 5F–H**). These results show that ILK antagonizes the repressive function of SUV39H1 and TRIM28 on viral gene transcription.

**Figure 6:**
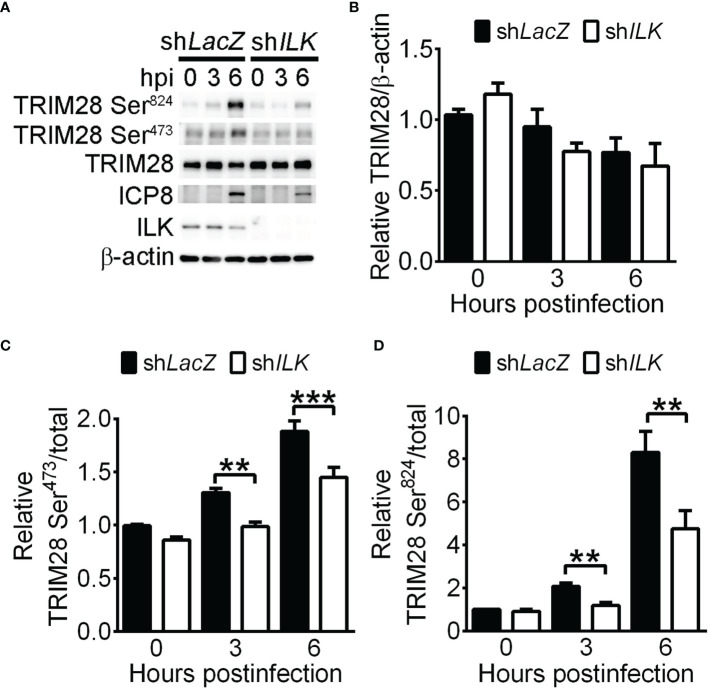
ILK facilitates TRIM28 phosphorylation on serine 473 and 824 in HSV-1-infected cells. The representative western blots of indicated proteins **(A)** and quantitative results **(B–D)** of indicated proteins in control (sh*LacZ*) and ILK (sh*ILK*) knockdown cells infected with HSV-1 at indicated hours postinfection are shown. The levels of TRIM28 to β-actin and phosphorylated TRIM28 to total TRIM28 in control cells at 0 hours postinfection were set as 1. Data represent mean + SEM (error bar). **p < 0.01, ***p < 0.001 *via* Student *t* test.

It has been shown that the phosphorylation of TRIM28 on serine positions 473 (Ser^473^) and 824 (Ser^824^) reflects a reduced binding of TRIM28 on heterochromatin proteins that results in chromatin relaxation and target gene activation (Ziv et al., 2006; Chang et al., 2008; Goodarzi et al., 2011; Lee et al., 2012; Ayrapetov et al., 2014). Therefore, we determined if TRIM28 phosphorylation is reduced in ILK knockdown cells. HSV-1 infection increased TRIM28 Ser^473^ and Ser^824^ levels in control cells at 3 and 6 hpi (**Figure 6A**). ILK knockdown decreased TRIM28 Ser^473^ and Ser^824^ levels without affecting total TRIM28 levels (**Figures 6A–D**), suggesting ILK promotes TRIM28 phosphorylation to reduce its binding on HSV-1 promoters.

### ILK Inhibitor Reduces HSV-1 Replication in Cells and Mice

**Figure 7:**
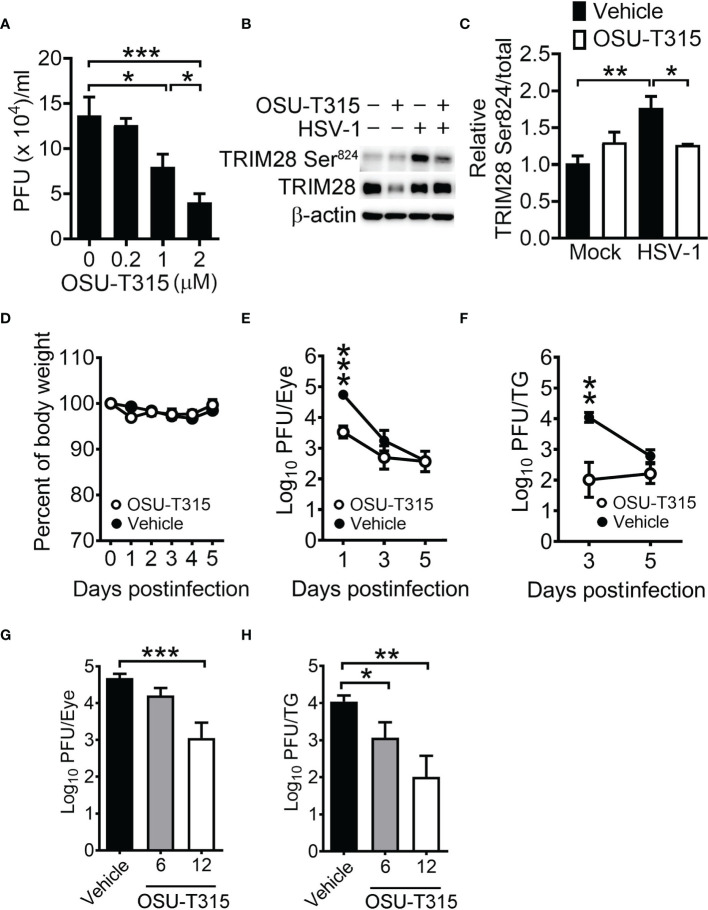
ILK inhibitor reduces HSV-1 replication in cells and mice. **(A)** The viral yields at 24 hours postinfection in SK-N-SH cells infected with HSV-1 (MOI = 0.01) and treated with the indicated concentration of OSU-T315 one hour after infection are shown. **(B, C)** The representative western blots **(B)** and relative levels **(C)** of indicated proteins at 3 hours postinfection in mock- (-) or HSV-1-infected (+) cells (MOI = 1) treated with vehicle (-) or 2 µM OSU-T315 (+) are shown. The level of TRIM28 Ser^824^ normalized to TRIM28 in mock-infected cells treated with vehicle was set as 1. **(D–F)** C57BL/6J mice were infected with 2 × 10^5^ PFU of KOS and treated with vehicle (n = 7) or 12 mg/kg OSU-T315 (n = 7). The relative body weight **(D)** and HSV-1 titers in the eyes **(E)** and trigeminal ganglia (TG; **F**) are shown. The body weight of mice on the day of infection was set as 1. **(G, H)** The HSV-1 titers in the eyes on 1 day postinfection **(G)** and trigeminal ganglia on 3 days postinfection **(H)** of infected mice treated with vehicle (n = 4) or the indicated concentration of OSU-T315 (n = 5 in each group) are shown. Data represent mean ± or + SEM (error bar). *p < 0.05, **p < 0.01, ***p < 0.001 *via* Student *t* test in panel C and Mann-Whiteny *U* test in the rest panels.

To determine if the ILK inhibitor suppresses TRIM28 phosphorylation and HSV-1 replication, we treated HSV-1-infected SN-K-SH cells with OSU-T315 after 1 hour of infection. The half-maximal inhibitory concentration (IC_50_) of OSU-T315 to inhibit cell proliferation is 4.4 µM. OSU-T315 treatment reduced the mean HSV-1 yields in cells in a manner dependent on doses (**Figure 7A**) and TRIM28 Ser^824^ levels (**Figures 7B, C**). We next address the significance of ILK in HSV-1 replication *in vivo* using a murine model, in which mouse cornea were scarified and inoculated with HSV-1 The bodyweight remained unchanged in infected mice treated with OSU-T315 (12 mg/kg; **Figure 7D**), suggesting OSU-T315 did not induce evident side effects. The HSV-1 titers were lower in the eyes and trigeminal ganglia of OSU-T315-treated mice than those of vehicle-treated mice (**Figures 7E, F**). We further treated mice with 6 or 12 mg/kg OSU-T315 and determined HSV-1 titers in mouse eyes on 1 day postinfection and trigeminal ganglia on 3 days postinfection, when the HSV-1 titers were highest in the tissues. We found that OSU-T315 decreased HSV-1 replication in mouse tissues in a manner dependent on doses (**Figures 7G, H**).

## Discussion

The H3K9me3 is associated with HSV-1 DNA early after infection to suppress lytic gene expression (Liang et al., 2009; Liang et al., 2013; Johnson et al., 2014; Orzalli et al., 2016; Cabral et al., 2018; Merkl and Knipe, 2019). However, little is known about the factors and mechanism in trimethylating H3K9 in HSV-1 infection. Here, we reveal that the H3K9 methyltransferase SUV39H1 and transcription corepressor TRIM28 bound to and repressed the activation of HSV-1 IE and E promoters. We further demonstrate that ILK reduced the binding of SUV39H1, TRIM28, and H3K9me3 on viral promoters to increase HSV-1 replication. Treatment of ILK inhibitor, OSU-T315, decreased HSV-1 replication in cells and mice. To our knowledge, this is the first report showing the enhancing role of ILK in HSV-1 infection by antagonizing SUV39H1- and TRIM28-mediated H3K9me3 accumulation on viral DNA.

We demonstrate that ILK enhanced HSV-1 replication. ILK has been shown to facilitate the coxsackie B3 virus and Rift Valley Fever virus infections by activating Akt (Esfandiarei et al., 2006; Pinkham et al., 2017). ILK increases the hepatitis C virus infection by inhibiting IFN-α-induced PKR activation and ISG15 expression (Kuwashiro et al., 2018). Akt has been shown to promote HSV-1 infection by enhancing viral entry (Cheshenko et al., 2013; Musarrat et al., 2018) and suppressing cGAS-STING-dependent type I IFN production (Seo et al., 2015), suggesting a pro-viral role of ILK in HSV-1 infection by activating Akt or repressing type I IFN production. However, neither HSV-1 entry and penetration nor the expression of *IFNB*, *CXCL10*, and *ISG15* were affected by ILK knockdown. Ectopic expression of ILK restored HSV-1 replication regardless of Akt activation, indicating that ILK-mediated Akt activation promotes RNA virus infections but is dispensable for HSV-1 replication. Instead, we demonstrate that ILK enhanced HSV-1 replication by reducing the levels of H3K9me3 on viral promoters and in replication compartments. ILK knockdown decreased the levels of viral gene transcripts and DNA duplication.

VP16 and ICP0 activate HSV-1 lytic gene expression by promoting H3K9me3 removal (Liang et al., 2009; Liang et al., 2013; Johnson et al., 2014; Orzalli et al., 2016; Cabral et al., 2018; Merkl and Knipe, 2019). As the absence of ICP0 failed to abrogate ILK-enhanced HSV-1 replication, and HSV-1 promoter activities were suppressed in uninfected ILK knockdown cells, the results indicate that ILK regulates intrinsic cellular proteins instead of viral proteins to facilitate HSV-1 replication. Although the regulation of H3K9me3 in HSV-1 infection is extensively studied, the redundant functions of multiple H3K9 methyltransferases make it challenging to identify the specific enzyme trimethylating H3K9 on HSV-1 promoters. The point is supported by our finding that SUV39H1 knockdown failed to further increase HSV-1 replication in control SK-N-SH cells (1.9 ± 0.4 and 2.2 ± 0.5 PFU(×10^5^)/ml in cells treated with scrambled and SUV39H1-specific siRNA, respectively) and by Cabral et al. showing that knockdown of SETDB1 or SETDB1 plus SUV39H1 fails to enhance HSV-1 replication in human fibroblasts (Cabral et al., 2021). By studying the responsible factors for H3K9me3 accumulation in ILK knockdown cells, we reveal that the SUV39H1, SUV39H2, and TRIM28 bound HSV-1 IE and E promoters. ILK knockdown increased SUV39H1 and TRIM28 without affecting SUV39H2 binding to HSV-1 promoters. Knocking down SUV39H1 or TRIM28 restored viral gene transcription and viral yields in ILK knockdown cells, confirming that ILK acts upstream of SUV39H1 and TRIM28 to enhance HSV-1 infection. The differential regulation of SUV39H1 and H2 by ILK has not been reported before and might be associated with their tissue-specific functions, which require further investigation. Previous studies showed that SUV39H1 facilitates cellular heterochromatin formation *via* trimethylating H3K9. The accumulated H3K9me3 recruits TRIM28 to enhance H4K20 trimethylation *via* SUV420H (Schotta et al., 2004; Jang et al., 2018; Wang et al., 2019). We also detected a limited increase in H4K20me3 on HSV-1 IE and E promoters in ILK knockdown cells, suggesting that SUV39H1 and TRIM28 facilitate H3K9me3 accumulation on HSV-1 genomes in a process resembling cellular heterochromatin formation. Furthermore, the nucleosome remodeler CHD3 has been shown to bind the SUV39H1-TRIM28 complex (Lee et al., 2012). CHD3 represses HSV-1 IE gene transcription by decreasing viral DNA accessibility (Arbuckle and Kristie, 2014). Notably, we show that SUV39H1 and TRIM28 binding were increased in ILK knockdown cells, suggesting that ILK antagonizes SUV39H1 and TRIM28 binding to impair SUV39H1-mediated H3K9 trimethylation and CHD3 recruitment leading to increased HSV-1 DNA accessibility and gene transcription. The SUV39H1 and TRIM28 display no DNA-binding ability. In cells, TRIM28 is guided by KRAB zinc-finger proteins to specific DNA regions (Peng et al., 2000). The nuclear DNA sensor IFI16 binds and facilitates heterochromatin marks accumulation on HSV-1 DNA *via* an unknown mechanism (Johnson et al., 2014; Merkl and Knipe, 2019). In addition, IFI16 is responsible for SUV39H1 recruitment to viral DNA during *de novo* infection and latency of Kaposi’s sarcoma-associated herpesvirus (Roy et al., 2019). Further studies are required to determine if IFI16 or other DNA-binding proteins can recruit the SUV39H1-TRIM28 complex to HSV-1 DNA.

We demonstrate that ILK enhanced HSV-1 replication by antagonizing TRIM28- and SUV39H1-mediated repression. Phosphorylated TRIM28 exhibits a reduced ability to interact with heterochromatin proteins, including SUV39H1 and CHD3, resulting in chromatin relaxation and target gene activation (Ziv et al., 2006; Chang et al., 2008; Goodarzi et al., 2011; Lee et al., 2012; Ayrapetov et al., 2014). In herpesvirus infections, TRIM28 phosphorylation either on serine 473 or 824 triggers the lytic gene expression of latent cytomegalovirus (Rauwel et al., 2015) and Epstein-Barr virus (Li et al., 2017). In our studies, HSV-1 infection increased the phosphorylation of TRIM28 on serine 473 and 824 in cells. Notably, ILK knockdown decreased the level of HSV-1-induced TRIM28 phosphorylation on both sites, suggesting that ILK enhances TRIM28 phosphorylation to impair its interaction with SUV39H1 and chromatin remodeler, such as CHD3. Further studies are needed to clarify if ILK phosphorylates TRIM28 directly or indirectly by inducing kinase cascades. Previous studies showed that ILK shuttles into the cell nucleus (Chun et al., 2005; Acconcia et al., 2007; Nakrieko et al., 2008). Nuclear ILK increases DNA synthesis in keratinocytes (Nakrieko et al., 2008) and represses the *CNKSR3* gene expression in MCF-7 cells (Acconcia et al., 2007), suggesting that nuclear ILK could regulate DNA accessibility *via* unclear mechanisms. The E359K mutant impairs ILK localization to focal adhesions (Nikolopoulos and Turner, 2002), while has limited effect on ILK nuclear import (Nakrieko et al., 2008). Our studies show that ILK E359K mutant restored HSV-1 replication as wild-type ILK did, suggesting that ILK might function in the cell nucleus to regulate the assembly of SUV39H1-TRIM28 complex on HSV-1 DNA. To test if HSV-1 increases ILK import into the cell nucleus, we detected the ILK levels in the cell nucleus and cytoplasm of mock- and HSV-1-infected control cells at 6 hpi by western blot analysis. The comparable levels of ILK were detected in the cytoplasm (1.1 ± 0.08 and 1.2 ± 0.3 in mock- and HSV-1-infected cells, respectively) and cell nucleus (1.2 ± 0.2 and 1.3 ± 0.5 in mock- and HSV-1-infected cells, respectively) of control cells, showing that HSV-1 infection failed to affect ILK import to the cell nucleus. Further studies are required to address how and where ILK promotes TRIM28 phosphorylation in HSV-1 infection.

TRIM28 promotes the latency maintenance of cytomegalovirus (Rauwel et al., 2015), Epstein-Barr virus (Li et al., 2017), and human immunodeficiency virus (Ma et al., 2019) and repression of endogenous retrotransposons (Rowe et al., 2010; Fasching et al., 2015; Brattas et al., 2017) by accumulating H3K9me3 on DNA of viral genomes or endogenous retrotransposons. TRIM28 knockdown or TRIM28 phosphorylation stimulates viral reactivation from latency and transcription activation of endogenous retrotransposons. Specifically, we show that ILK promoted TRIM28 phosphorylation in HSV-1 lytic infection. Taken together, ILK has the potential to reactivate dormant viral genomes in cells by reducing TRIM28-mediated accumulation of heterochromatin marks. Moreover, targeting ILK can be a broad-spectrum antiviral strategy for RNA and DNA viruses, especially for DNA virus infections controlled by histone modifications.

## Data Availability Statement

The original contributions presented in the study are included in the article/**Supplementary Material**. Further inquiries can be directed to the corresponding author.

## Ethics Statement

The animal study was reviewed and approved by Institutional Animal Care and Use Committee in National Cheng Kung University with the approval number 108101.

## Author Contributions

M-ST and S-HC contribute to the work equally. M-ST performed the in vitro experiments and wrote the first draft of the manuscript. S-HC provided materials and designed and performed the in vivo experiments. C-PC provided materials, technical support, and critical suggestions. Y-LH performed experiments and provided technical support. L-CW designed, performed, and supervised the experiments and wrote the manuscript. All authors contributed to the article and approved the submitted version.

## Funding

This work is funded by the Ministry of Science and Technology (MOST 108-2321-B-214-001-MY3) and by I-Shou University (ISU109-S-02), Taiwan, to L-CW.

## Conflict of Interest

The authors declare that the research was conducted in the absence of any commercial or financial relationships that could be construed as a potential conflict of interest.

## Publisher’s Note

All claims expressed in this article are solely those of the authors and do not necessarily represent those of their affiliated organizations, or those of the publisher, the editors and the reviewers. Any product that may be evaluated in this article, or claim that may be made by its manufacturer, is not guaranteed or endorsed by the publisher.
