# Supplementary material for: Integrin-Linked Kinase Reduces H3K9 Trimethylation to Enhance Herpes Simplex Virus 1 Replication
Source: Front Cell Infect Microbiol. 2022 Mar 8;12:814307. doi: 10.3389/fcimb.2022.814307 (PMC8957879; doi:10.3389/fcimb.2022.814307)
Supplement: Supplementary file 1 [file DataSheet_1.pdf]

Supplementary Material

**Primers for plasmid construction**		
FW-ICP0p	ATCTAGCTCGAGGTTGGGCCCCCAAATCGG	
RE-ICP0p	ATCTAGAAGCTTCGCTCCGAGTGCCGAGG	
ILK-HindIII	ATCTAGAAGCTTGCCACCATGGATTACAAGGATG	
ILK-EcoRI	ATCTAGGAATTCTTAACGCGTCTTGTCCTGCATC	
ILK-FW-359	GTAGCCCCCAAAGCTCTGCAG	
ILK-RE-359	CTGCAGAGCTTTGGGGGCTAC	
**Primers for cellular and viral DNA**		
**Gene**	**Forward**	**Reverse**
tk	CTTAACAGCGTCAACAGCGTGCCG	CCAAAGAGGTGCGGGAGTTT
ACTB	CTATCCCTGTACGCCTCTGG	TGGTGGTGAAGCTGTAGCC
**Primers for viral and cellular gene promoters**		
ICP0	CGCGGGTCGCTCAATGAAC	GCCCGGCCCCCGATT
ICP4	GCCCCTGGGACTATATGAGC	GCGTCTGACGGTCTGTCTCT
ICP8	GAGACCGGGGTTGGGGAATGAATC	CCCCGGGGGTTGTCTGTGAAGG
GAPDH	CAGGCGCCCAATACGACCAAAATC	TTCGACAGTCAGTCAGCCGCATCTTCTT
D4Z4	CTCAGCGAGGAAGAATACCG	ACCGGGCCTAGACCTAGAAG
**Primers for cellular and viral RNA**		
ICP0	CCCACTATCAGGTACACCAGCTT	CTGCGCTGCGACACCTT
ICP4	CGACACGGATCCACGACCC	GATCCCCCTCCCGCGCTTCGTCCG
ICP8	GTCGTTACCGAGGGCTTCAA	GTTACCTTGTCCGAGCCTCC
ICP27	GCATCCTTCGTGTTTGTCATTCTG	GCATCTTCTCTCCGACCCCG
ACTB	CATGTACGTTGCTATCCAGGC	CTCCTTAATGTCACGCACGAT
ILK	ATGGAACCCTGAACAAACACT	AGCACATTTGGATGCGAGAAA
IFNB	GCTTGGATTCCTACAAAGAAGCA	ATAGATGGTCAATGCGGCGTC
CXCL10	GTGGCATTCAAGGAGTACCTC	TGATGGCCTTCGATTCTGGATT
ISG15	TGGTGGACAAATGCGACGAA	CAGGCGCAGATTCATGAAC
SUV39H1	GTCATGGAGTACGTGGGAGAG	CCTGACGGTCGTAGATCTGG
SUV39H2	ATTGATAACCTCGATACTCGTCTT	TCTCCAGAACCTTTCATTTGATAA

Supplementary Table 1. Sequences of primers.

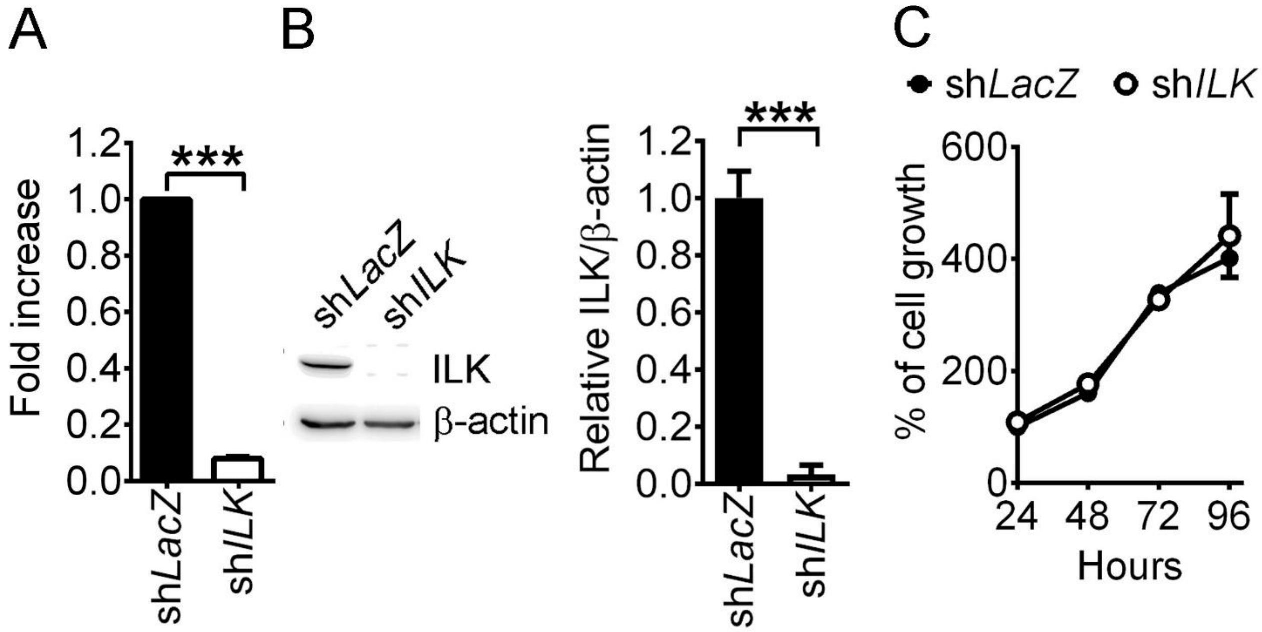

Supplementary Figure 1. ILK knockdown fails to affect cell viability. **(A)** The relative mRNA levels of *ILK* in control (sh*LacZ*) and ILK knockdown (sh*ILK*) cells are shown. The mRNA level of *ILK* normalized to *ACTB* in control cells was set as 1. **(B)** The representative western blots of indicated proteins (left panel) and relative ILK levels (right panel) in control and ILK knockdown cells are shown. The level of ILK normalized to β-actin in control cells was set as 1. **(C)** The proliferation rates of control and ILK knockdown cells at indicated time points are shown. The viability of control cells at 24 hours after seeding was set as 100%. Data represent mean + or ± SEM (error bar). ***p < 0.001 via Student t test.

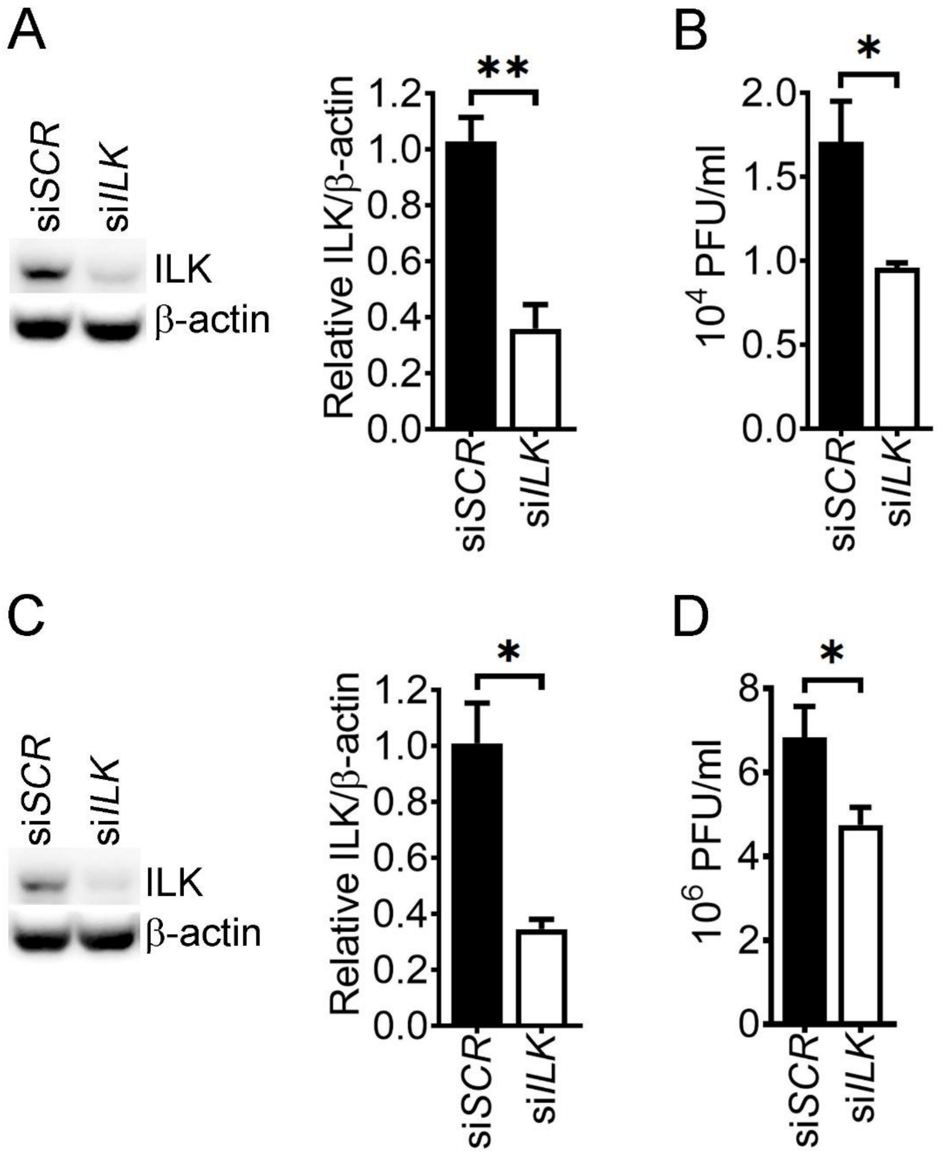

Supplementary Figure 2. ILK knockdown by siRNA reduces HSV-1 replication. **(A and C)** The representative western blots of indicated proteins (left panel) and quantitative results (right panel) of SK-N-SH cells **(A)** and ARPE-19 cells **(C)** transfected with scrambled siRNA (si*SCR*) or siRNA specific to *ILK* (si*ILK*) are shown. The level of ILK normalized to β-actin in control cells was set as 1. **(B and D)** The viral yields in HSV-1-infected (MOI = 0.01) SK-N-SH cells **(B)** and ARPE-19 cells **(D)** transfected with indicated siRNA at 24 hours postinfection are shown. Data represent mean + SEM (error bar). *$p < 0.05$, **$p < 0.01$ via Student t test in panel A and C via Mann-Whitney U test in panel B and D.

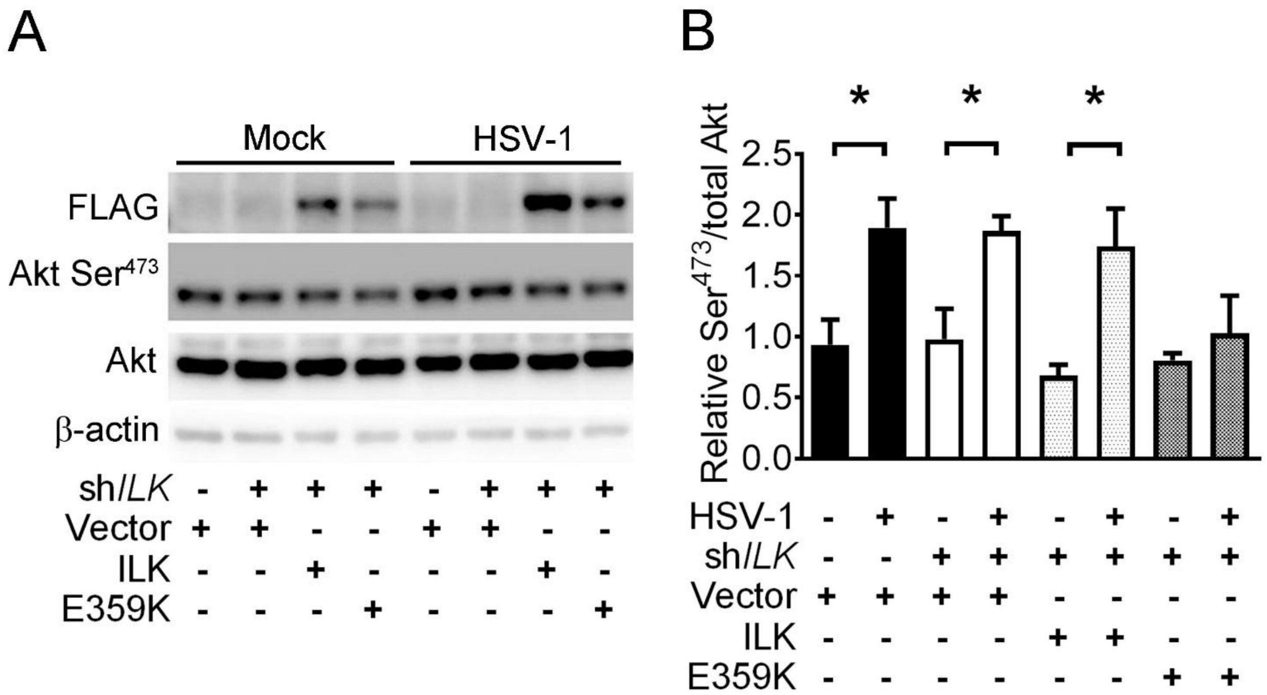

Supplementary Figure 3. The levels of phosphorylated Akt in control and ILK knockdown cells without or with ectopic expression of ILK. **(A and B)** The representative western blots of indicated proteins **(A)** and quantitative results **(B)** of phosphorylated Akt on serine 473 (Ser^{473}) in the control (-) and ILK knockdown cells (+) transfected with a control vector (Vector) or vector expressing wild-type ILK (ILK) or ILK E359K mutant tagged with 3×FLAG and infected with HSV-1 (MOI = 0.01) for 24 hours are shown. The level of Akt Ser^{473} normalized to Akt in uninfected control cells transfected with a control vector was set as 1. Data represent mean + SEM (error bar). *$p < 0.05$ via Student *t* test.

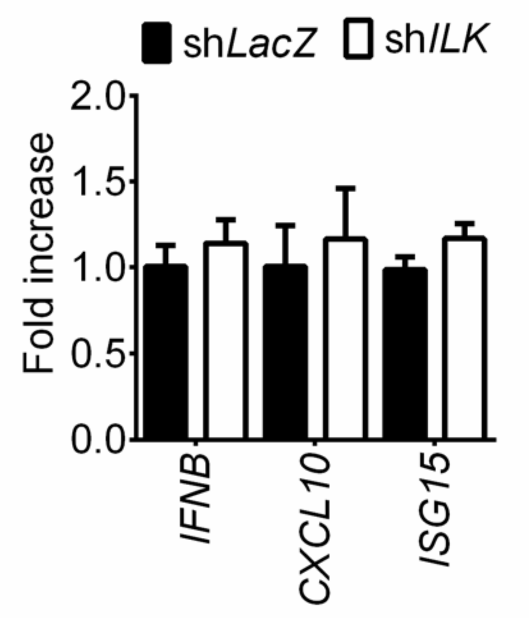

Supplementary Figure 4. ILK knockdown fails to affect the levels of *IFNB*, *CXLC10*, and *ISG15*. The relative mRNA levels of indicated genes in the control (sh*LacZ*) and ILK knockdown (sh*ILK*) cells infected with HSV-1 for 6 hours are shown. The mRNA levels of indicated genes normalized to *ACTB* mRNA in control cells were set as 1. Data represent mean + SEM (error bar). ***$p < 0.001$ via Student *t* test.

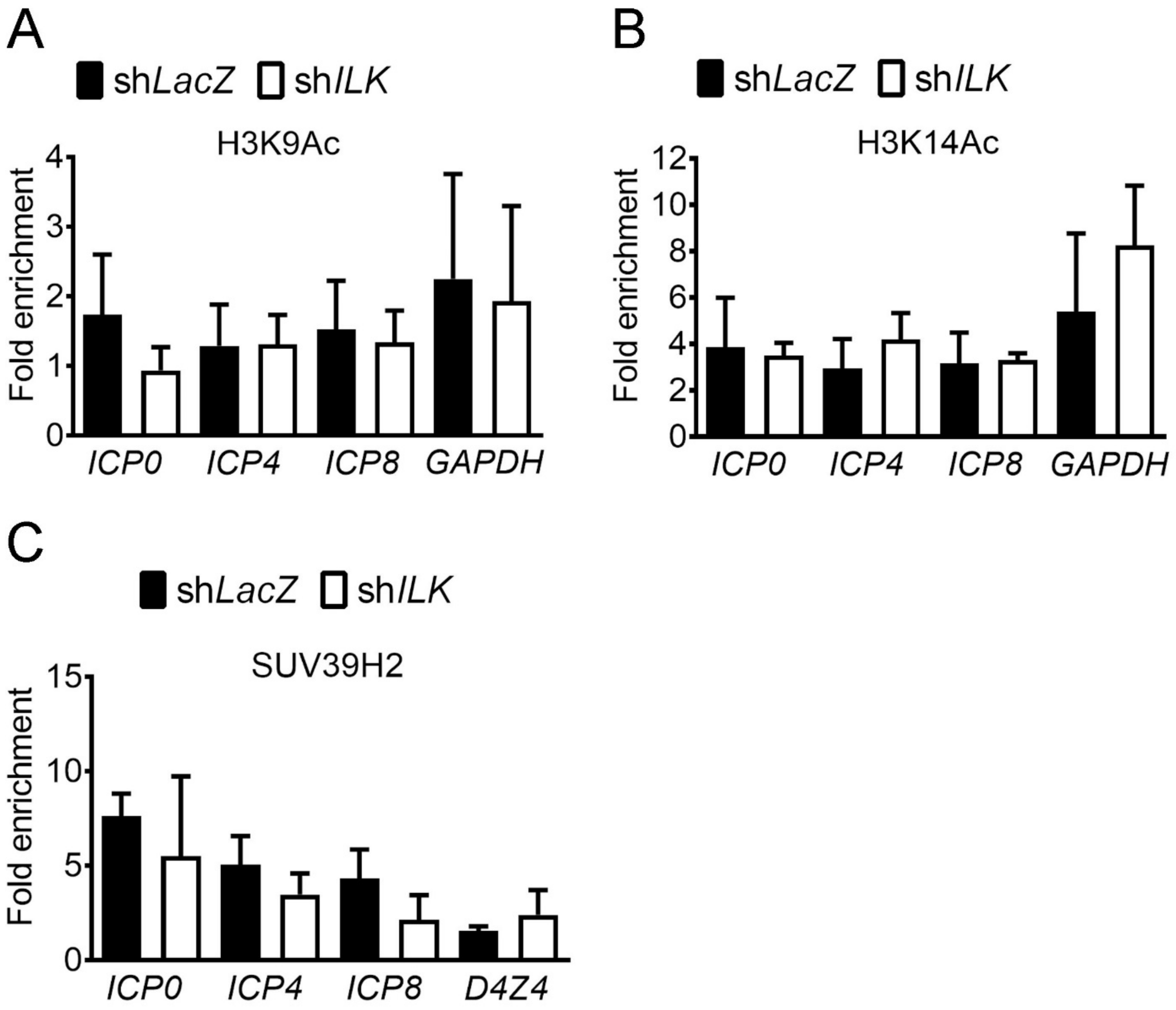

Supplementary Figure 5. ILK fails to affect the levels of acetylated H3K9 and H3K14 and SUV39H2 in HSV-1-infected cells. **(A to C)** The relative levels of acetylated (Ac) H3K9 **(A)**, H3K14Ac **(B)**, and SUV39H2 **(C)** associated with *ICP0*, *ICP4*, and *ICP8* promoters in control (sh*LacZ*) and ILK knockdown (sh*ILK*) infected with HSV-1 at 3 hours postinfection are shown. The *GAPDH* promoter region serves as a positive control for H3K9Ac and H3K14Ac binding, and the non-satellite repeat, *D4Z4*, is a positive control of SUV39H2 binding. Fold enrichment was calculated as the fraction of DNA immunoprecipitated by the specific antibody/input normalized to the control antibody/input. Data represent mean + SEM (error bar).

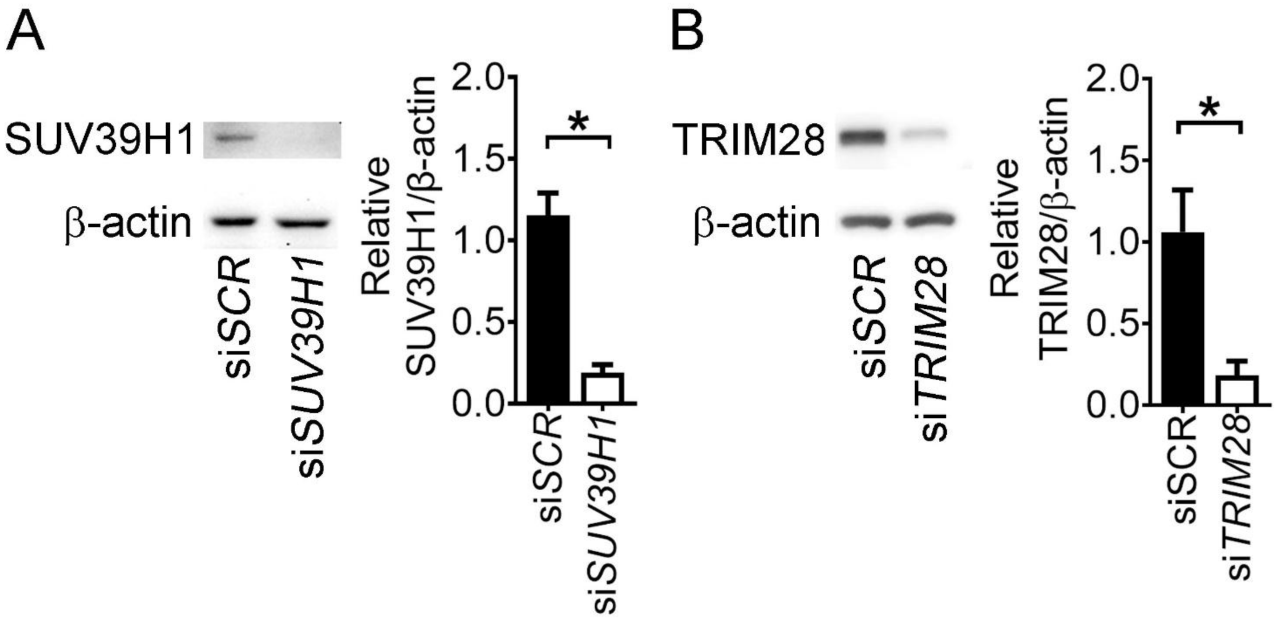

Supplementary Figure 6. SUV39H1 and TRIM29 knockdown efficacy in ILK knockdown cells. The representative western blots of indicated proteins (left panel) and quantitative results (right panel) of ILK knockdown SK-N-SH cells transfected with scrambled siRNA (si*SCR*) or siRNA specific to SUV39H1 (si*SUV39H1*; **A**) or siRNA specific to TRIM28 (si*TRIM28*; **B**) are shown. The level of indicated protein to β-actin in cells with siSCR was set as 1. Data represent mean + SEM (error bar). *$p < 0.05$ via Student t test.
